# Establishment of a PCR analysis method for canine BRCA2

**DOI:** 10.1186/1756-0500-5-173

**Published:** 2012-04-03

**Authors:** Yasunaga Yoshikawa, Masami Morimatsu, Kazuhiko Ochiai, Kento Okuda, Takahiro Taoda, Seishiro Chikazawa, Asako Shimamura, Toshinori Omi, Makoto Bonkobara, Koichi Orino, Kiyotaka Watanabe

**Affiliations:** 1 Laboratory of Veterinary Biochemistry, School of Veterinary Medicine, Kitasato University, Aomori 034-8628, Japan; 2 Division of Disease Model Innovation, Institute for Genetic Medicine, Hokkaido University, Sapporo 060-0815, Japan; 3 Department of Basic Science, School of Veterinary Nursing and Technology, Nippon Veterinary and Life Science University, Tokyo 180-8602, Japan; 4 Department of Small Animal Surgery 2, School of Veterinary Medicine, Kitasato University, Aomori 034-8628, Japan; 5 Department of Small Animal Internal Medicine 2, School of Veterinary Medicine, Kitasato University, Aomori 034-8628, Japan; 6 Department of Veterinary Science, School of Veterinary Medicine, Nippon Veterinary and Life Science University, Tokyo 180-8602, Japan

## Abstract

**Background:**

Mammary tumors are the most common tumor type in both human and canine females. In women, carriers of mutations in BRCA2, a tumor suppressor gene product, have a higher risk of breast cancer. Canine *BRCA2 *has also been suggested to have a relationship with mammary tumors. However, clearly deleterious BRCA2 mutations have not been identified in any canine mammary tumors, as appropriate methods to detect mutations or a consensus BRCA2 sequence have not been reported.

**Findings:**

For amplification and sequencing of BRCA2, we designed 14 and 20 PCR primer sets corresponding to the BRCA2 open reading frame (ORF) and all 27 exons, respectively, including exon-intron boundaries of the canine BRCA2 regions, respectively. To define the consensus canine BRCA2 ORF sequence, we used established methods to sequence the full-length canine BRCA2 ORF sequence from two ovaries and a testis obtained from individual healthy mongrel dogs and partially sequence BRCA2 genomic sequences in 20-56 tumor-free dogs, each aged over 6 years. Subsequently, we compared these sequences and seven previously reported sequences, and defined the most common base sequences as the consensus canine BRCA2 ORF sequence. Moreover, we established a detection method for identifying splicing variants. Unexpectedly, we also identified novel splicing variants in normal testes during establishment of these methods.

**Conclusions:**

The present analysis methods for determining the BRCA2 base sequence and for detecting BRCA2 splicing variants and the BRCA2 ORF consensus sequence are useful for better understanding the relationship between canine BRCA2 mutation status and cancer risk.

## Findings

Mammary tumors are the most common tumor type in both human and canine females, constituting about half of all tumors in female dogs [1-4]. Furthermore, approximately half of canine mammary tumors are malignant [5,6]. In humans, heritable breast cancers have been linked with mutations in the breast cancer susceptibility gene *BRCA2*. Genetic analysis, including detection of deleterious mutations and splicing variants, to identify BRCA2 mutation carriers is strongly advocated, as the lifetime risk of breast cancer is high (81-88%) for females carrying a BRCA2 mutation [7,8].

In a recent study, it was suggested that the canine *BRCA2 *gene locus is associated with mammary tumors based on single nucleotide polymorphism analysis of an intronic marker [9,10]. Consistent with this notion, we previously showed that loss of heterozygosity, which is one of the mechanisms of *BRCA2 *inactivation, was present in a mammary tumor [11]. Canine BRCA2 missense mutations have also been reported in mammary tumors [11-13]. However, clearly deleterious mutations in the canine BRCA2 sequence have not been identified in mammary tumors due to the lack of appropriate methods to detect such mutations. Furthermore, a full-length consensus canine BRCA2 open reading frame (ORF) sequence has not been defined, as full-length canine BRCA2 has only been identified in a single sample [14].

**Figure 1:**
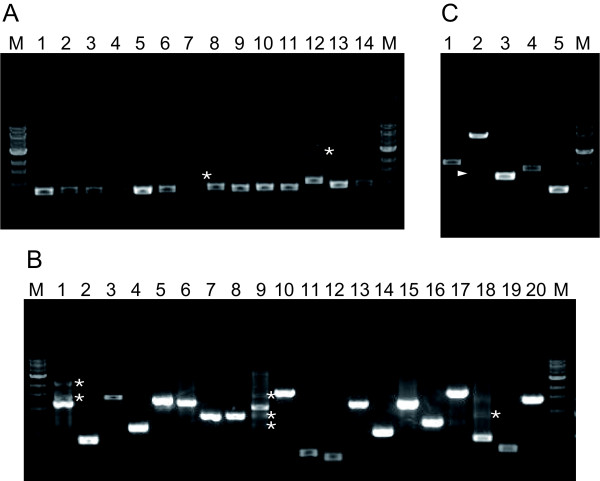
**PCR products amplified by each primer set**. (**A**) cDNA samples prepared from total RNA of each mammary gland were amplified. (**B**) Genomic DNA from each mammary gland was amplified. (**C**) Splicing variants of the cDNA from total RNA of the mammary gland and testis were amplified. Primer sets for each lane are shown in Table 1. The "M" indicates the molecular size marker (1-kbp DNA ladder; New England Biolabs). Arrowhead and "*" indicate novel BRCA2 transcript and non-specific PCR products, respectively.

Determination of the base sequence of *BRCA2 *in a tumor sample and of this sequence comparison with the *BRCA2 *consensus sequence is the most standard method for detecting mutations in tumor samples in humans. During the course of our present study, one study reported the mutation analysis of full-length of canine BRCA2, but they used many primer sets (about 50 sets) and amplified sequence only from genomic DNA [15]. To establish a more efficient mutation analysis method for cDNA and genomic DNA that requires fewer primer sets, we designed 14 and 20 primer sets in order to sequence the BRCA2 ORF and all 27 exons, respectively, including the exon-intron boundaries of the canine BRCA2 regions. All PCR targets were successfully amplified, and were sufficient to determine DNA base sequences (Figure 1A and 1B).

**Figure 2:**
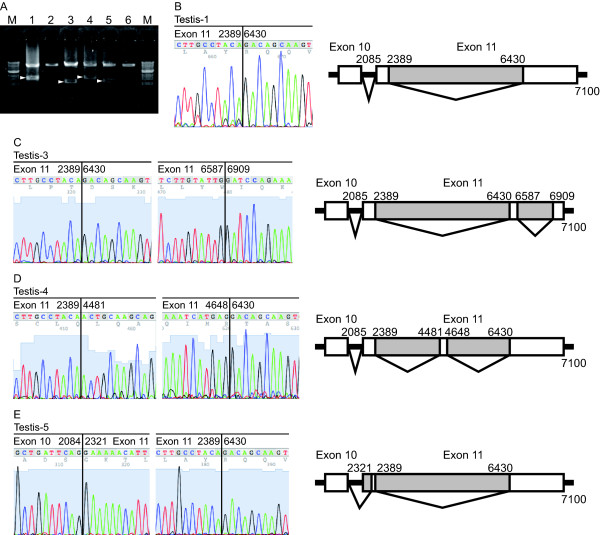
**Identification of splicing variants within exon 11 in normal testes**. The splicing variants identified in normal testes lacked a large portion of exon 11. (**A**) To confirm the presence of a novel BRCA2 transcript, splicing variants of the cDNA from total RNA of the testes were amplified using nested PCR. (B-E) Electropherogram showing the direct sequencing data and overview of the novel BRCA2 transcript that lacked nucleotides 2390 to 6429 (**B**), 2390 to 6429 and 6588 to 6908 (**C**), 2390 to 3380, and 4649 to 6429 (**D**) and 2085 to 2321 and 2390 to 6429 (E) from Testis-1, -3, -4, and -5, respectively. The Testis-1 was the same sample used to generate the data in Figure 1C. Primer sets for each lane are shown in Table 1. The "M" indicates the molecular size marker (1-kbp DNA ladder). Arrowheads indicate novel BRCA2 transcripts.

Some splicing variants of tumor suppressor genes (e.g., *BRCA2*) in tumor tissue have been associated with tumorigenesis because these splicing variants often lead to frameshift mutations [16,17]. Thus, we next designed five primer sets for detecting splicing variants from cDNA (Figure 1C). All PCR targets were successfully amplified, and the predicted sizes of PCR products were confirmed. During the establishment of this method, we unexpectedly identified splicing variants between exon 10 and exon 14 in normal testes (Figure 1C and 2). These transcripts skipped most of exon 11, leading to frameshift mutations (Figure 2).

To define the consensus canine BRCA2 ORF, we sequenced the full-length canine BRCA2 ORF in two ovaries and a testis obtained from individual healthy mongrel dogs using the method described here. We identified six single nucleotide variations (516 T > C, 1366 T > G, 2428 T > G, 2609A > C, 4481A > C and 8257 T > C) and two insertion/deletion polymorphisms (7126ins/delGTT and 10204ins/delAAA) (Accession numbers: AB622997, AB622998 and AB622999). None of these variations resulted in nonsense or frameshift mutations. To determine the most common base sequences and generate a consensus canine BRCA2 ORF sequence, we compared these three new sequences (six alleles) and the seven previously reported sequences (Accession numbers: AB043895.5, NC_006607.2, Z75664 and FJ464397-FJ464400) (Table 2). The four variations (516 T (103I), 2428 T (740 G), 4481A (1425 T), and 8257 T (2683I)) could be defined as consensus base sequences, but the other four variations (1366 T > G, 2609A > C (K801Q), 7126ins/delGTT, and 10204ins/delAAA) could not be defined as such because the frequencies between the major and minor alleles in each variation were nearly identical. We therefore sequenced these four variations in genomic DNA from 20-23 normal blood samples from tumor-free dogs aged over 6 years; the methods described here were used (Tables 3 and 4). We finally defined the most common base sequences as the consensus canine BRCA2 ORF sequence (Table 2). The 10204insAAA variation was consensus sequence in dogs, but in four miniature Dachshunds this variation was determined to be a minor variation (allele ratio; del:ins = 6:2, Table 4). To confirm the consensus sequence in miniature Dachshunds, we sequenced BRCA2 DNA from an additional 32 blood samples, and the assembled allele ratio was del:ins = 30:42 (Table 4).

We established a PCR analysis method for canine BRCA2 in order to determine the base sequence from cDNA and genomic DNA, and to detect splicing variants. We identified novel splicing variants in normal canine testes. The functions of these splicing variants were not assessed in this study; nevertheless, these results indicated that the established method was a useful tool for detecting splicing variants.

We also defined the consensus sequence using methods established and described here. During the definition of the consensus BRCA2 ORF, we identified three novel (516 T > C, 2428 T > G, and 8257 T > C) and three reported (1366 T > G, 2609A > C and 4481A > C) single nucleotide variations and two reported insertion/deletion polymorphisms (7126ins/delGTT and 10204ins/delAAA) (Accession numbers: AB622997, AB622998 and AB622999) [11,12,15,18]. The variations 1366 T > G (C386W), 2609A > C (K801Q), 4481A > C (T1425P), and 10204ins/delAAA (M3332IV) are located in the histone acetyltransferase domain, the FANCG binding domain, BRC repeat 3, and nuclear localization signal 2, respectively [13,19-21]. The effects of these variations on BRCA2 function were not understood, with the exception of 10204insAAA; nuclear localization signal 2 harboring the 10204insAAA variation showed enhanced nuclear localization [13]. The other nonsynonymous variations were not located in previously known functional domains.

We identified four variations (1366 T > G, 2609A > C, 7126ins/delGTT, and 10204ins/delAAA), in which the allele frequency of minor variations in genomic DNA from normal blood samples was very high (28.5-37.5%). Such frequent variations in the *BRCA2 *gene have not been reported in other species. These highly frequent variations thus appear to be a canine BRCA2-specific feature, and should be considered when studying canine BRCA2. These four variations were found in the homozygous state in some blood samples from elderly tumor-free dogs. Homozygous mutations in BRCA2 are assumed to be embryonic-lethal mutations or responsible for Fanconi anemia, which is characterized by bone marrow failure, developmental abnormalities, and predisposition to cancer [22,23]. Thus, these four variations were probably neutral variations, although the 10204insAAA variation is reportedly a candidate malignant mutation in dogs [11].

In this study, we established a PCR analysis method and defined the consensus sequence of BRCA2 ORF to identify canine BRCA2 mutations. Using these methods, we are now able to perform BRCA2 mutation analysis and search for abnormal BRCA2 splicing variants from mammary tumors in dogs, as is done in human cases.

## Methods

### Specimens

Two ovaries (from two mongrel dogs), six testes (from a mongrel dog and five Beagles), a mammary gland (from a female Beagle) and 56 blood samples (Table 4) from tumor-free dogs were kindly provided by Dr. Takashi Kubo and Dr. Go Honda. All experimental procedures were approved by and conducted in accordance with the Guidelines for Institutional Laboratory Animal Care and Use of the School of Veterinary Medicine at Kitasato University, Japan (Approval Number: 11-065).

### Total RNA and genomic DNA extraction, and preparation of cDNA

Total RNA was isolated from ovaries, six testes, and one mammary gland, which each were stored in RNAlater solution (Life Technologies, Grand Island, NY), using a TRIzol and PureLink RNA micro kit (Life Technologies). First-strand cDNA was synthesized from 1-5 μg of total RNA using SuperScript III (Life Technologies). Genomic DNA samples were extracted using a Gentra Puregene tissue kit (Qiagen, Hilden, Germany).

### PCR and sequencing

For PCR amplification of the full-length canine BRCA2 ORF from cDNA and all 27 exons from genomic DNA, we designed 14 and 20 primer sets, respectively (Table 1). We also designed five primer sets to detect splicing variants and a primer sets to confirm a novel BRCA2 transcript that lacked most of exon 11 using nested PCR (Table 1). Each reaction mixture contained 0.1 μL of first-strand cDNA reaction products or 10-50 ng of genomic DNA as a template, each forward and reverse primer at 300 nM, 200 μM dNTPs, 0.02 U of KOD FX DNA polymerase (Toyobo, Japan), and 1× PCR buffer, which was supplied with the enzyme, in a total volume of 10 μL. PCR included one cycle of 2 min at 94°C, followed by 35 cycles of 10 s at 98°C, 30 s at the optimal temperature shown in Table 1 the optimal time shown in Table 1 at 68°C, and a final extension step of 7 min at 68°C. PCR products were treated with shrimp alkaline phosphatase (Affymetrix, Santa Clara, CA) and Exonuclease I (New England BioLabs, Beverley, MA) before sequencing, which was performed with the BigDye Terminator Cycle Sequencing kit Version 3.1 and a ABI PRISM 3100-Avant DNA sequencer (Life Technologies). Direct DNA sequencing was performed at least twice for each amplicon. When we attempted to define the consensus canine BRCA2 ORF sequence, two or three amplicons from each sample were sequenced. Because we detected only three electropherogram patterns among the PCR products with the insertion/deletion mutation sites, we were able to determine the heterozygous insertion/deletion mutations by direct sequencing (Additional file 1: Figure S1).

**Table 1:** Nucleotide base sequences of primers

	Primer sets	Forward	Reverse	Annealing temperature	Elongation time	Lane Number	Expected sizes
For amplification of cDNA	1	5'-GCGGCACCTCGGAAGGC-3'	5'-CCCCAAACTTTGACTTTTAGC-3'	60°C	1 min	Figure 1 A 1	834 bp

	2	5'-GATCGGTTTATCCCTTGTGGTC-3'	5'-CTTCAGGTTCTTTAAAGTTTGG-3'	60°C	1 min	Figure 1 A 2	865 bp

	3	5'-CTGAAGGGATGTCCAATGC-3'	5'-ATATTTTATATGATTCTTTTCCTC-3'	56.1°C	1 min	Figure 1 A 3	850 bp

	4	5'-CCAGTCTGTTAACTCCTAGC-3'	5'-GGATAATGTTCCTCAATATCTTTG-3'	60°C	1 min	Figure 1 A 4	826 bp

	5	5'-ACAGCTTCTAATAAAGAGATAAA AC-3'	5'-GCCGGCATTTATTATTTTTC-3'	56.1°C	1 min	Fig. 1 A 5	850 bp

	6	5'-GTTTCTCCTCAAGCAAATACAA-3'	5'-ATTTTTTACTTTGTCCAAAGATTCC-3'	60°C	1 min	Figure 1 A 6	873 bp

	7	5'-CTGATCCTGCAGCAAAGACC-3'	5'-GAAAAACCAATGTTTTTTCTCTCTC-3'	59.2°C	1 min	Figure 1A 7	908 bp

	8	5'-CATTCTAGTGAAGTGTATAATAA ATCAG-3'	5'-CTGTCCTAAATCCAGAGAAAGC-3'	50.8°C	1 min	Figure 1 A 8	919 bp

	9	5'-AGTATCACTTAAAGATAATGAAG AAC-3'	5'-CTTTTAGGATGCCGTCTGG-3'	50°C	1 min	Figure 1 A 9	887 bp

	10	5'-CCCCCAATTAAAAGAAACTTG-3'	5'-GCCAATTGTATTCCTTCTCC-3'	53.7°C	1 min	Figure 1 A 10	905 bp

	11	5'-CCTCTGCATGTTCTCATAAAC-3'	5'-GGGTATGCTCTTTGAACAACTAC-3'	60°C	1 min	Figure 1 A 11	886 bp

	12	5'-CATGGAGCAGAACTGGTAGG-3'	5'-GTGTAAGGTTTAATAATGTCTTCA-3'	50°C	1 min	Figure 1 A 12	1094 bp

	13	5'-CCTATCCCAAGTTTATCAGCC-3'	5'-CAGACACAAGTTGATGTTCTCC-3'	60°C	1 min	Figure 1 A 13	959 bp

	14	5'-GAAGGCATTTCAGCCACCACG-3'	5'-CAATCACACTAGAATCATAAAAAGG-3'	60°C	1 min	Figure 1 A 14	978 bp

For amplification of genomic DNA	exon 1-2	5'-GCCCCCTGCCCAGAACCC-3'	5'-CTTTTCAGCAAGCATGCACAGTTACG-3'	60°C	2 min	Figure 1 B 1	1193 bp

	exon 3	5'-CTACAGTCAAAATGTCAAGCG-3'	5'-CACAATTAACAATAGATCTGGGAG-3'	60°C	1 min	Figure 1 B 2	430 bp

	exon 4-7	5'-ATAAGAATAAAAACTTCCAGAGAATG-3'	5'-ATTATCTTTTCATATATTCTTTTTGTC-3'	60°C	2 min	Figure 1 B 3	1384 bp

	exon 8-9	5'-GTAGTATATGTGACTTTTGATGTCTG-3'	5'-GGAAAAGCAATGTATTTTCTCTTT-3'	60°C	2 min	Figure 1 B 4	615 bp

	exon 10	5'-CTTTAAATACTGCCTTATGGGCTA-3'	5'-GTCACCATCCCTAAAACTATATGC-3'	60°C	2 min	Figure 1 B 5	1311 bp

	exon 11-a	5'-GTCACTTTGTGTCTTCATGC-3'	5'-GGATAATGTTCCTCAATATCTTTG-3'	56.4°C	2 min	Figure 1 B 6	1246 bp

	exon 11-b(same as primer set 5)	5'-ACAGCTTCTAATAAAGAGATAAAAC-3'	5'-GCCGGCATTTATTATTTTTC-3'	56.4°C	1 min	Figure 1 B 7	850 bp

	exon 11-c(same as primer set 6)	5'-GTTTCTCCTCAAGCAAATACAA-3'	5'-GATTTTTTACTTTGTCCAAAGATTCC-3'	60°C	1 min	Figure 1 B 8	873 bp

	exon 11-d(same as primer set 7)	5'-CTGATCCTGCAGCAAAGACC-3'	5'-GAAAAACCAATGTTTTTTCTCTCTC-3'	59.2°C	1 min	Figure 1 B 9	908 bp

	exon 11-e	5'-CATTCTAGTGAAGTGTATAATAAATCAG-3'	5'-ATTCCCCTAAACTATACATAAAAG-3'	56.4°C	2 min	Figure 1 B 10	1720 bp

	exon 12	5'-CAATTCTTTAGTTTTAAAAAATGG GC-3	5'- GAAAAAGCTTAGAAAAAGAACTTAAAAAATAC-3'	59.2°C	1 min	Figure 1 B 11	275 bp

	exon 13	5'- GTAAATGTTTATAATGTGTAATATACAGGC-3'	5'-CTGTACCTTCCCTACACTATATTAGTAG-3'	60°C	1 min	Figure 1 B 12	230 bp

	exon 14-15	5'-CCAAACTTAAATATTTTCTCCTC-3'	5'-CAGGGATCCCAGTCTATTC-3'	60°C	2 min	Figure 1 B 13	1213 bp

	exon 16	5'-GCAGCAAACCCTTGAATGTAG-3'	5'-GTCAGGTGAACCGTAATGTG-3'	60°C	1 min	Figure 1 B 14	552 bp

	exon 17-8	5'- GGTCTTGTACAGTGTAGTGTTTG-3'	5'-GTTTTTAAGCAATGGAGCATC-3'	59.2°C	2 min	Figure 1 B 15	1258 bp

	exon 19-20	5'- CCATCATGTTTAAATTGAAGTCTC-3'	5'-CAATTACAGAGGTTAAATCAGAAGCC-3'	59.2°C	2 min	Figure 1 B 16	739 bp

	exon 21-24	5'-CTCGATATTTGATTCACCAGC-3'	5'-CAACAGTCCCTTCCTAACCC-3'	60°C	2 min	Figure 1 B 17	1739 bp

	exon 25	5'- CAGTATCACTTTTTCTACATTTTG GTC-3'	5'-CCCAATTTTCACAGAAGCCAC-3'	59.2°C	1 min	Figure 1 B 18	471 bp

	exon 26	5'-GGCTTCCATAGATGTTAGATG-3'	5'-GGACAACTTGGGATCATTTGC-3'	50.8°C	1 min	Figure 1 B 19	337 bp

	exon 27	5'- GCTAAATTGCTGATGTTTTCTAC-3'	5'-CTGCTGAGTCCTCTAATAAGGC-3'	60°C	2 min	Figure 1 B 20	1437 bp

	exon 25	5'- CAGTATCACTTTTTCTACATTTTGGTC-3'	5'-CCCAATTTTCACAGAAGCCAC-3'	59.2°C	1 min	Figure 1 B 18	471 bp

	exon 26	5'-GGCTTCCATAGATGTTAGATG-3'	5'-GGACAACTTGGGATCATTTGC-3'	50.8°C	1 min	Figure 1 B 19	337 bp

	exon 27	5'- GCTAAATTGCTGATGTTTTCTAC-3'	5'-CTGCTGAGTCCTCTAATAAGGC-3'	60°C	2 min	Figure 1 B 20	1437 bp

For detection of splicing variants	exon 1-11	5'-CGAATTTGTTAGCCGTCTCC-3'	5'-GGATCCTGAGATATTATTTTATTATTAG-3'	60°C	2.5 min	Figure 1 C 1	2118 bp

	exon 10-14	5'-CTGAAGGGATGTCCAATGC-3'	5'-GAAATTTGGATTCTGTATTTCTTG-3'	58°C	6 min	Figure 1 C 2	5594 bp and 1554 bp

	exon 11-18	5'-CTTCCTGTGAAAACAAATATAG-3'	5'-GCTGATCTTCTGCTTTTATC-3'	50.8°C	2 min	Figure 1 C 3	1417 bp

	exon 15-25	5'-CCTCTGCATGTTCTCATAAAC-3'	5'-GTGTAAGGTTTAATAATGTCTTCA-3'	60°C	2 min	Figure 1 C 4	1759 bp

	exon 24-27 (same as primer set 13)	5'-CCTATCCCAAGTTTATCAGCC-3'	5'-CAGACACAAGTTGATGTTCTCC-3'	60°C	2 min	Figure 1 C 5	959 bp

For nested PCR of the transcripts lacking most of exon 11	exon 10-13(1735-7280)	5'-GTTCTCAAATAATATGACTAATCCAAAC-3'	5'-GTTCCTCAGTTGTGCGAAAG-3'	58°C	6 min	Figure 2 A	5546 bp and 1506 bp, 1185 bp, 1674 bp or 1270 bp

For DNA sequence	cB2 seq1	5'-CAATAGAGGTGTTTTCTCCATC-3'					

	cB2 seq2	5'-GGATCCTGAGATATTATTTTATTATTAG-3'					5546 bp and 1506 bp, 1185 bp, 1674 bp or 1270 bp

	cB2 seq3	5'-CCAGCTTTGTCTTTAACCAG-3'					

	cB2 seq4	5'-CTGTGTGACCACTTTCACTATC-3'					

	cB2 seq5	5'-CCCTCCTTCATAAACTGGC-3'					

	cB2 seq6	5'-CTTTCTGAGAGGCATGATCTG-3'					

	cB2 seq7	5'-GCATGGCAAGTGTCTGATTTAC-3'					

	cB2 seq8	5'-GTGAACAAACTTCACAACTTAACC-3'					

	cB2 seq9	5'-GCTGATCTTCTGCTTTTATC-3'					

	cB2 seq10	5'-GGTATGTTTTACAATGATGC-3'					

	cB2 ex14 R (exon)	5'-CTAAAGGTTCTTTTTCATTCTTTG-3'					

	cB2 ex15 F	5'- GCTTTTTAAATGTTACATGGAGG-3'					

	cB2 ex17 R	5'-GTACCAGTCAGGGATGTGAG-3'					

	cB2 ex18 F (exon)	5'-ATATGATGTGGAAATTGATAAAA G-3'					

	cB2 ex22F	5'-CTTTTTAAAGGGATTCATTTACAG TGG-3'					

	cB2 ex23 F (exon)	5'-CCATCACCAGATTTATATTCCC-3'					

	cB2 ex26 R (exon)	5'-CAGAAATTTATTTCCTATGCC-3'					

	cB2 ex23 F (exon)	5'-CCATCACCAGATTTATATTCCC-3'					

	cB2 ex26 R (exon)	5'-CAGAAATTTATTTCCTATGCC-3'					

**Table 2:** Comparison between our sequences from the canine BRCA2 open reading frame with registered sequences

Nucletide location^a^		516 T > C	1366 T > G	2428 T > G	2609A > C	4481A > C	7126delGTT	8257 T > C	10204insAAA
Amino acid		I103T	C386W	Silent	K801Q	T1425P	2307delL	Silent	M3332IK

Coding exon		3	10	11	11	11	12	18	27

Novel or reported variation		Novel	Reported	Novel	Reported	Reported	Reported	Novel	Reported

Present resequencing results^b^									

Full length	Ovary 1	T/C	G/G	T/G	C/A	A/C	ins/del	T/C	ins/ins

	Ovary 2	T/T	G/G	T/T	C/C	A/A	del/del	T/T	ins/ins

	Testis	T/T	G/G	T/T	C/C	A/A	del/del	T/T	ins/ins

Partial	Genome	N. D.	T:G =	N. D.	A:C =	N. D.	ins:del =	N. D.	del:ins =
									
			12:30		29:15		25:15		17:29

Registered sequences^c^									

Ochiai et al.	Testis	T	T	T	A	A	ins	T	del

Genome project	Genome	T	T	T	A	A	ins	T	del

Bignell et al.	Genome	-	-	T	A	A	-	-	-

Hsu et al.	Mammary gl.	-	-	T	A	A	-	-	-

Total allele frequency		T:C =	T:G =	T:G =	A:C =	A:C =	ins:del =	T:C =	del:ins =
		
		7:1	14:36	12:1	37:20	12:1	28:20	7:1	19:35

Consensus sequence		516 T	1366 G	2428 T	2609A	4481A	7126insGTT	8257 T	10204insAAA
	
		(103I)	(386 W)	(740 G)	(801 K)	(1425 T)	(2307insL)	(2638I)	(3332IK)

N. D., not determined.

^a^Nucleotide and amino acid location is based on AB043895.5

^b^Full-length sequence was determined by cDNA sequencing (Accession number; AB622997, AB622998 and AB622999). When frequencies of major and minor alleles were nearly equal or were inconsistent with reported sequences, alleles were further analyzed by partial sequencing of blood genome DNA from 20-23 dogs (Table 3 and 4).

^c^Sequence from one dog was regarded as one allele because allele type analyses have not been described in these reports. The study by Hsu et al. examined three dogs, while others studied only one dog. Accession numbers for sequences reported by Ochiai et al., the Genome Project, Bignell et al. and Hsu et al. are AB043895.5, NC_006607.2, Z75664 and FJ464397-FJ464400, respectively.

**Table 3:** Genotype frequency of four variations in normal blood samples

Nucletide location^a^	1366 T > G		2609A > C		7126delGTT		10204insAAA	
Amino acid^a^	C386W		K801Q		2307delL		M3332IK	

Coding exon	10		11		12		27	

Genotype frequency	1366 T homozygosity	2/21	2609A homozygosity	10/22	insGTT homozygosity	9/20	delAAA homozygosity	5/23
	
	1366 G homozygosity	11/21	2609 C homozygosity	3/22	delGTT homozygosity	4/20	insAAA homozygosity	11/23
	
	Heterozygosity	8/21	Heterozygosity	9/22	Heterozygosity	7/20	Heterozygosity	7/23

^a^Nucleotide and amino acid locations are based on AB043895.5

**Table 4:** Information of blood samples and allele type of the four frequently found variations

Sample name	Sex	Year	Breed	Nucletide location^a^			
				**1366 T > G**	**2609A > C**	**7126delGTT**	**10204insAAA**

K-1	Male	7	Beagle	G/G	C/C	del/del	ins/ins

K-2	Male	9	Labrador retriever	G/G	A/C	ins/del	ins/ins

K-3	Female	9	Mongrel dog	G/T	A/A	ins/ins	del/ins

K-4	Male	10	Bichon Frise	G/T	A/A	ins/ins	del/ins

K-5	Female	12	Pomeranian	G/G	A/A	ins/ins	ins/ins

K-6	Female	12	Puli	G/T	A/A	ins/ins	del/del

K-7	Female	6	Puli	G/T	A/A	ins/ins	del/ins

K-8	Male	10	Miniature Dachshund	G/T	A/C	ins/del	del/ins

K-9	Male	8	Miniature Dachshund	G/T	A/C	ins/del	del/del

K-10	Male	8	Miniature Dachshund	T/T	A/A	ins/ins	del/del

K-11	Female	7	Papillon	G/G	C/C	del/del	ins/ins

K-12	Female	7	Mongrel dog	G/G	A/A	ins/ins	-

K-13	Female	12	Miniature Pinscher	T/T	A/A	ins/ins	del/del

K-14	Male	14	mongrel dog	G/G	A/C	ins/del	ins/ins

K-15	Male	14	mongrel dog	G/G	A/A	-	ins/ins

K-17	Female	7	Papillon	-	C/C	-	ins/ins

K-19	Female	9	Mongrel dog	G/G	A/A	ins/ins	del/ins

K-20	Male	10	Mongrel dog	G/T	A/C	ins/del	ins/ins

K-21	Female	7	Mongrel dog	G/G	A/C	-	ins/ins

K-23	Male	10	Mongrel dog	G/G	A/C	-	del/ins

K-26	Male	15	Mongrel dog	G/G	A/C	ins/del	ins/ins

K-27	Female	9	Miniature Dachshund	-	A/C	ins/del	del/ins

K-28	Male	6	Cavalier King Charles Spaniel	G/T	-	del/del	ins/ins

K-29	Male	12	Mongrel dog	-	-	del/del	del/del

MD-1	Male	10	Miniature Dachshund	-	-	-	del/del

MD-2	Male	7	Miniature Dachshund	-	-	-	del/del

MD-3	Male	6	Miniature Dachshund	-	-	-	ins/ins

MD-4	Male	10	Miniature Dachshund	-	-	-	del/ins

MD-5	Male	12	Miniature Dachshund	-	-	-	del/ins

MD-6	Female	7	Miniature Dachshund	-	-	-	del/ins

MD-7	Male	12	Miniature Dachshund	-	-	-	del/del

MD-8	Male	11	Miniature Dachshund	-	-	-	del/ins

MD-9	Female	9	Miniature Dachshund	-	-	-	del/ins

MD-10	Male	9	Miniature Dachshund	-	-	-	ins/ins

MD-11	Male	10	Miniature Dachshund	-	-	-	del/ins

MD-12	Female	14	Miniature Dachshund	-	-	-	del/ins

MD-13	Male	9	Miniature Dachshund	-	-	-	ins/ins

MD-14	Male	6	Miniature Dachshund	-	-	-	ins/ins

MD-15	Male	6	Miniature Dachshund	-	-	-	ins/ins

MD-16	Female	7	Miniature Dachshund	-	-	-	del/ins

MD-17	Male	8	Miniature Dachshund	-	-	-	del/del

MD-18	Male	12	Miniature Dachshund	-	-	-	del/ins

MD-19	Male	10	Miniature Dachshund	-	-	-	ins/ins

MD-20	Male	6	Miniature Dachshund	-	-	-	ins/ins

MD-21	Female	6	Miniature Dachshund	-	-	-	del/ins

MD-22	Male	10	Miniature Dachshund	-	-	-	ins/ins

MD-23	Female	10	Miniature Dachshund	-	-	-	del/ins

MD-24	Female	7	Miniature Dachshund	-	-	-	del/ins

MD-25	Female	7	Miniature Dachshund	-	-	-	ins/ins

MD-26	Male	8	Miniature Dachshund	-	-	-	ins/ins

MD-27	Female	9	Miniature Dachshund	-	-	-	del/ins

MD-28	Male	9	Miniature Dachshund	-	-	-	ins/ins

MD-29	Male	8	Miniature Dachshund	-	-	-	del/ins

MD-30	Male	8	Miniature Dachshund	-	-	-	del/ins

MD-31	Male	9	Miniature Dachshund	-	-	-	ins/ins

MD-32	Female	12	Miniature Dachshund	-	-	-	del/ins

^a^Nucleotide and amino acid locations are based on AB043895.5

"-" indicates not determined.

## Competing interests

The authors declare that they have no competing interests.

## Authors' contributions

YY outlined the design of and coordinated the study, performed the experiments, and drafted the manuscript. MM, K. Ochiai, K. Orino, and WK participated in the design of the study and interpretation of the data and helped to draft the manuscript. K. Okuda, TT, SC, SA, TO, and MB performed several experiments.

## Supplementary Material

Additional file 1 **Figure S1**. Example of an electropherogram by direct sequencing from PCR products having the insertion/deletion mutation (7126ins/delGTT). Click here for file
