# Supplementary material for: Establishment of a PCR analysis method for canine BRCA2
Source: BMC Res Notes. 2012 Apr 3;5:173. doi: 10.1186/1756-0500-5-173 (PMC3355023; doi:10.1186/1756-0500-5-173)
Supplement: Additional file 1 — Figure S1. Example of an electropherogram by direct sequencing from PCR products having the insertion/deletion mutation (7126ins/delGTT). [file 1756-0500-5-173-S1.PDF]

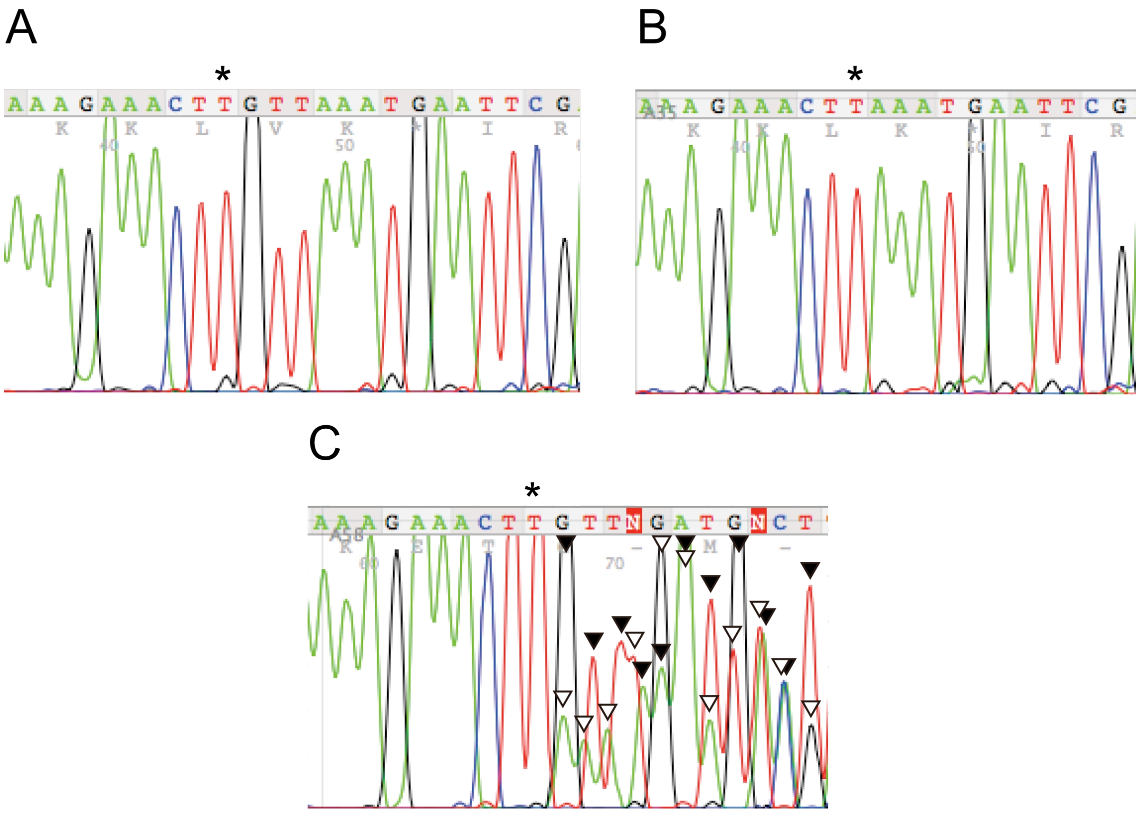

Supplementary Figure 1. Example of an electropherogram by direct sequencing from PCR products having the insertion/deletion mutation (7126ins/delGTT).

(A) and (B) Electropherograms of the PCR products with 7126insGTT (A) and 7126delGTT (B). We could determine the homozygous insertion or deletion mutation. (C) Electropherogram by direct sequencing of the PCR products with heterozygous 7126ins/delGTT. We detected overlapped electropherograms of the PCR products with 7126insGTT and 7126delGTT. Closed and open arrowheads indicate the electropherogram from 7126insGTT (7126-GTTAAATGAAT-7136) and 7126delGTT (7126-AAATGAATTCG-7136), respectively. Electropherograms that showed the same pattern as this electropherogram were determined to be from PCR products with heterozygous 7126ins/delGTT, because the presence of other types of insertion/deletion mutations would show different electropherogram patterns. Asterisk indicates the nucleotide of 7125T.
